# In education we trust: on handling the COVID-19 Pandemic in the Swedish welfare state

**DOI:** 10.1007/s11618-021-01001-y

**Published:** 2021-03-03

**Authors:** Sverker Lindblad, Anders Lindqvist, Caroline Runesdotter, Gun-Britt Wärvik

**Affiliations:** Department of Education and Special Education, University of Gothenburg, SE 405 30 Gothenburg, Sweden

**Keywords:** Pandemic, School lockdown, Self-governance, Social trust, Welfare state education, Pandemie, Schulschließung, Selbstverwaltung, Soziales Vertrauen, Wohlfahrtsstaat, Bildung

## Abstract

Keeping schools open was an active strategy in Sweden to meet the threats of the COVID-19 pandemic. In this article we analyze how a collection of welfare state agents with different tasks, resources and interests in interaction formed an assemblage in their responses to the pandemic and how education thereby became part of a strategy to keep the society going. The inquiries concern what this tells us about education as framed and constrained as a part of society. Our observations are based on statements presented by the government and public agencies, mass media and websites. We identified an assemblage of interwoven agents such as institutions, laws, regulations and recommendations, pandemic manuals, statistics and media. All these were brought together by actions and ideas to handle a pandemic when there were no preventive vaccines. The overarching principle was to educate the population to competent actions in dealing with the pandemic. To keep schools open was part of that principle combined with caretaking ambitions. This assemblage looked like a centralistic machine but it was not; risks were pushed back to local authorities and schools. In conclusion, we note that education is vital in the overarching strategy to deal with the pandemic in Sweden in terms of trust in people and governmentality.

## Abstract (de)

Im Umgang mit der Corona-Pandemie entschied sich die schwedische Regierung bewusst dafür, Schulen offen zu halten. Im vorliegenden Artikel analysieren wir, wie ausgewählte Entscheider im Wohlfahrtsstaat mit unterschiedlichen Aufgaben, Ressourcen und Interessen interagierten und zu einer gemeinsamen Strategie in Bezug auf die Pandemie gelangten. Bildung wurde dabei zum Element einer Gesamtstrategie mit dem Ziel, die Gesellschaft funktionsfähig zu halten. Wir gehen der Frage nach, was dies über Bildung im Kontext ihrer Rahmung und Beschränkung durch die Gesellschaft aussagt. Unsere Analysen beruhen auf Verlautbarungen der Regierung und öffentlicher Einrichtungen, Beiträgen in Massenmedien und Websites. Wir konnten ein Netz von Handlungsträgern und deren Verflechtungen in Institutionen, Gesetze, Regelungen und Empfehlungen, Handreichungen zu Pandemien, Statistiken und Medien identifizieren. Die Ideen und Aktionen beziehen sich auf die Phase, in der es noch keine Schutzimpfungen gegen die Corona-Pandemie gab. Dem Ganzen übergeordnet war die Leitidee, die Bevölkerung zum kompetenten Umgang mit der Pandemie zu erziehen. Die Offenhaltung des Schulbetriebs war ein wichtiger Pfeiler in der Strategie, verbunden mit dem Gedanken der Fürsorge. Was auf den ersten Blick wie ein zentralistisch organisierter Apparat anmutet, war es bei genauerem Hinsehen nicht: Risiken wurden doch auf die lokale Ebene und die Schulen verlagert. Zusammenfassend stellen wir fest, dass der Bildung in der schwedischen Strategie des Umgangs mit der Corona-Pandemie eine Schlüsselrolle zukommt – bei einer lenkenden Rolle der Regierung und zugleich Vertrauen in die Bevölkerung.

## Introduction

Sweden’s state epidemiologist Dr. Anders Tegnell stated that “We have [to be] able to keep the schools open” when interviewed by a British web channel in July 2020, adding that they think it “… is extremely important.”^1^ Tegnell is talking as a civil servant at the Public Health Agency of Sweden. He is referring to the compulsory comprehensive school in Sweden (primary and lower secondary levels) and to the fact that the Swedish government followed the recommendation of the agency on how to mitigate the COVID-19 pandemic.^2^ This pandemic, caused by a novel coronavirus, hit Swedish society hard. The factors that were important in the mitigation strategies were trust in social distancing, travel restrictions, improved personal hygiene among citizens, and comparatively little lockdowns. Thus, the responses for regulating the pandemic were, to a large extent, based on self-governance but were combined with repressive and financial measures.

In this article, we describe and analyze the actions and reactions in Sweden to the COVID-19 pandemic. Our focus is on education as a part of the welfare state and its state apparatuses in interaction with other agents. We ask how the education apparatus has responded to the COVID-19 crisis and why it has responded in such a way. We have neither the goal to assess this topic in terms of success or failure nor to defend the way the COVID-19 pandemic is being handled in Sweden. We are instead interested in analyzing the responses to the crisis in order to understand why they were carried out under the preconditions that were at hand, assuming that agents in other contexts also did the best they could but perhaps differently (e.g., Nóvoa and Yarif Mashal 2003). The basic idea is that the COVID-19 pandemic hit Swedish society, turning it out of normality, and, by this action, made the practices and agendas for agents, institutions, and their interactions visible for analysis.

Welfare state education is situated in a complex collection of interacting parts with different tasks, resources, and interests responding to the threats of COVID-19 when the crisis hit Sweden. In what ways is education a part of these responses, for what reasons, and what contexts? Keeping schools open was, we argue, an active pandemic strategy formulated in interaction with a collection of agents and networks that, under normal conditions, would be less visible. This study is significant for two reasons. First, it contributes to knowledge on educational tasks and strategies when dealing with pandemics of different kinds and under different preconditions. Second, it enriches our understanding of education as a part of a society, capturing how education is interwoven into different societal contexts. Given these, we need to deal with education in a societal framework or in terms of systems theory. As will be argued below, we turn into a more inductive position to identify the parts at work in a particular constellation and, through this, determine how such parts act and interact in the development of responses to the pandemic.

## Notes on previous studies

Since the beginning of mankind, human beings have been fighting against deadly contagious infections. A classic work on this topic is William McNeill’s *Plagues and People *in which he states that contagious diseases have the power to not only infiltrate human cells but also interact with and alter cultural and societal ecosystems, such as ideologies, politics, poverty, religious traditions, and wars (McNeill 2010 [1976]). However, research on societal responses to pandemics has focused, to a large extent, on medical analyses and mitigating aspects, such as antiviral strategies, and not on education (e.g., Ferguson et al. 2006).

Few historical studies are available on the Spanish influenza in 1918–1920 and its effects on schools. A small study regarding a debate on the closing of schools in the Vancouver area points out how such a decision became politicized (Andrews 1977). Should the children be protected from the school as a place for spreading the disease, or does the school instead offer a shelter *from* the disease, including a social shelter? Swedish studies on the Spanish influenza have not had a specific focus on schools but mention locally decided school closures when the number of influenza cases was raising (Åman 1990; Holmberg 2017). These studies tell us that pandemic control was connected with children’s economic position in society (family patterns, child labor, etc.) and with changes in the school system in a society in which schools had come to be seen as important for children. They also indicate that schools became possible non-pharmaceutical measures for regulating the pandemic in different parts of the world, even if knowledge about the virus during this time was lacking and even if international communication occurred much more slowly than it does today.

Educational challenges and responses have been the focus of some studies on more recent pandemics, mostly considering pandemic transmissions and school lockdowns, presenting contextual dependencies for lockdown strategies (e.g., Cauchemez et al. 2008; Vynnycky and Edmunds 2008). There are also studies on alternative educational measures or strategies, for instance, distance education as a substitute for classroom instruction (e.g., Williamson et al. 2020). Added to these are studies on how to deal with pandemics, such as AIDS or the current COVID-19 (see Reimers and Schleicher 2020).

To our knowledge, there is little research on educational responses to pandemics in terms of the emergence of interacting but autonomous parts dealing with these threats (On risks, see e.g., Brown 2020). What we find, however, are studies on lockdowns, as mentioned above, and epidemiological approaches to minimize the overall damages of mitigating strategies. Examples of this are research reviews used by the Public Health Agency of Sweden (Public Health Agency of Sweden 2020).

Moving to the second reason for the current study—considering education as interwoven in societal contexts—we turn into a classic field of studies in education that often deals with the ways in which education adjusts to societal powers (e.g., Bourdieu and Passeron 1990/1970), and how education is formed by changing societal structures (e.g., Durkheim 2013 [1902]). In relation to pandemics, we identify demands of self-governance by culturally competent subjects formed by the functioning of a social subsystem or persons who could act and interact in certain ways in the societal context of an ongoing pandemic.^3^ However, we are more interested in how education is responding to societal crises, as is presented by Ian Hunter in his study of schooling as a 17th-century innovation made to counteract population destruction.^4^ Thus, an interesting topic in the field of educational research is how education is made to respond in a crisis in an unknown situation.

## Points of departure

A starting point for this study is that COVID-19 turned into a societal threat that demanded joint or coordinated efforts from welfare state apparatuses amid a confused situation. We are analyzing these efforts by different agents and institutions—what did this mean to education, and what did education do under the pandemic? To deal with this emergent and complex problem, we need to capture how constellations of different parts are acting and how their efforts are eventually coordinated to deal with the pandemic at the same time as they are fulfilling their original tasks. In short, our inquiry is about a society out of normality because of the pandemic. To our understanding, this means that we cannot take notions of systems or subsystems and their boundaries for granted. Instead, conducting inquiries in an inductive way is reasonable in an attempt to capture a collection of different parts in the responses to the pandemic and how these responses interact. Furthermore, this disorder would presumably make some parts and practices visible in a way that they had not been otherwise.

Given this consideration, we took as a point of departure the attempt to identify and analyze a collection of different parts interwoven by the efforts to deal with the COVID-19 pandemic, which fits with assemblage theory (Deleuze and Guattari 1980) and, more precisely, a reception by DeLanda (2016). An assemblage:

is a multiplicity which is made up of many heterogenous terms and which establishes liaisons, relations between them, across ages, sexes and reigns—different natures. Thus, the assemblage’s only unity is that of a co-functioning: it is a symbiosis, a ‘sympathy’. (DeLanda 2016, p. 13)

In short, the parts of an assemblage are not uniform, but they are actively related to one another. Such relations can be internal, which means they are based on joint qualities inside the parts, or external, which means they are based on contingencies (DeLanda 2016, p. 14). We regard this approach as an open and systematic way to capture the meaning and impact of the pandemic and the different ways to understand how societies are dealing with this threat, for instance, in terms of coding (how the identities of parts are determined) and territorialization (homogenization and defining boundaries), such as the interrelations between different state apparatuses and ways of commanding different agents.

The general research problem, therefore, is to identify these parts and how they are (eventually) interrelated or, in other words, to capture an assemblage at work in responding to the pandemic. Our specific research problem is then to analyze education as an assemblage and its relations to the general pandemic assemblage.

Given that our research problem has broad framing, an initial analysis of our case is presented to us a set of parts and practices that we first identified in public discourses. The parts were:

- Government
- Public agencies
- Mass media
- International organizations
- Scientific communities

During our research, we identified more parts. We ask how education is interwoven in a assemblage—what parts have which relations with what consequences?

## Research questions

The focus of our research is to understand how and why education in Sweden responds to the pandemic. These responses are regarded as a part of a society that is trying to deal with an abnormal external threat imposed by a pandemic. In particular, we seek to answer the following questions:

- How is this case developing over time? What are the important events involved, and which agents appear in the making of responses by the education system to the pandemic? What decisions are made by which agents in such responses?
- How is education interacting with other parts of society? Which tasks are education dealing with in the societal responses to the pandemic, and which societal contexts are at work in organizing such responses?

We will discuss how education in the Swedish welfare state is functioning amid a societal crisis and what this tells us about education as framed and constrained as a part of society in its complexity.

## Reflections on data and observations

Data are based on observations of webpages and reports from international organizations, the Swedish government, national public authorities, press briefings, and media reporting. These observations are carried out during the pandemic, with little possibilities to conduct ethnographies because of pandemic restrictions. Such observations are, to a large extent, based on statements presented in mass media and the websites of different agents at work. This means that our data are produced by the agents we are observing—how they understand what is going on and what needs to be done with the crisis and how they present this in public. Therefore, we are using front stage observations, in which the actor is aware that they are being publicly observed as the data source.^5^ This was necessary because of the preconditions for the timing of the current research and our research interests. Given these restrictions, we will be careful in presenting sources and the contexts for the statements. We also plan to continue our study when the crisis is finally over.

The analysis focuses on *what moves *(c.f. Czarniawska and Sevón 1996). This concerns the identification and management of pandemic risks and uncertainties and how these, given the specific hierarchies of the Swedish administration and the cultural and social national contexts, came to be associated with and embrace school agents (c.f. Brown 2020)—what we call an assemblage (c.f. DeLanda 2016). We make a distinction between schools and the school system. The latter refers to administrative school agents (local and national authorities and ministers) and does not indicate a systemic analysis.

## Design of inquiries

Our research is built on a set of inquires of responses to the pandemic presented under the following three narratives:

- The challenge to keep schools open or closed
- Responses from the National Agency for Education
- The welfare state constitution and addressing a pandemic

The set of inquiries is used to capture how different agents come to constitute an assemblage in their responses to the pandemic and that made it possible to keep schools open or closed. Thus, each narrative presents different events, agents, responses, and interactions. We start with the schools, the overall focus of the three inquiries. Clearly, open or closed, the schools were heavily exposed and responded to challenges emanating from measures taken to regulate the pandemic. Thereafter, we turn to the National Agency for Education, one of the five public agencies in Sweden in the field of education. We have chosen the National Agency for Education, as it has a government commission that supports schools when dealing with a pandemic. The final inquiry concerns the Swedish welfare state constitution and its administration, which are vital in the strength and development of the demarcation of the assemblage. Therefore, the picture broadens to include agents outside the school sector; taken together, this can tell us how schools are acting as parts of an assemblage regulating the pandemic, parts that are interacting and non-reducible, and how they managed to be open or closed.

Table 1 presents an overview of agents, events and strategic responses to the pandemic.

**Table 1:** Agents, events, and strategic responses to the pandemic by the government and Ministry of Education (Gov), Public Health Agency (PHA), National Agency for Education (NAE), The Civil Contingencies Agency (CCA), County Medical Officer of Communicable Diseases (CMO), and laws

Date	Agent	Event	Strategic response
1 Feb	Communicable Diseases Act, Gov, PHA	COVID-19 classified as a disease that constitutes a public danger and a danger to society	Potentially repressive: Opened up for extraordinary communicable disease control measures
6 Mar	PHA	Start of the pedagogical program for infection control	Self-governing
11 Mar	WHO, PHA	COVID-19 classified as a pandemic	Potentially repressive: Pandemic plans fully activated and continuously revised
12 Mar	Gov, PHA, NAE	Preparation for school closure started	Potentially repressive: Local authorities could now close a school based on recommendations from the PHA or the CMO
13 Mar	Gov, PHA, NAE	New ordinance (SFS 2020: 115)	Potentially repressive: Preparations for new school legislation on the occasion of the spread of certain contagions
17 Mar	Gov, PHA, NAE	Closure of upper secondary schools and universities	Repressive: Local schools and institutions to transition to distance or remote education
19 Mar	Communicable Diseases Act, The School Law, Gov, NAE	New law on temporary changes in school activities (SFS 2020: 148)	Potentially repressive: Possible to temporarily stop school activities
20 Mar	Gov, CCA, NAE	New regulations from CCA	Supporting: Secure access to childcare for parents who have a job regarded as vital for society in case of school closure
22 Apr	Gov, NAE	NAE recommendation turned into a law	Potentially repressive: Secure access to education for children by paying attention to, investigating, and handling school absence
24 Apr	Gov, NAE, PHA	Government decision	Supporting: Possible (but not an obligation) to use distance or remote education in comprehensive education even if a school is open if many pupils and teachers need to stay at home when following the recommendation from the PHA
30 Apr	Gov, NAE	Government decision: A new commission in the NAE	Supporting: Produce and disseminate learning examples to support schools during the pandemic
15 Jun	PHA, Gov	New recommendation from the PHA	Self-governing: Upper secondary schools can go back to regular teaching and open up
16 Jun	Gov	New ordinance	Self-governing: If necessary, an upper secondary school may partly organize distance education to make it easier for it to follow the general recommendations from the PHA regarding public transportation

In addition to Table 1, we also identified a range of agents, such as schools, media, teachers’ trade unions, local authorities, and local emergency plans.

## The challenge to keep schools open or closed

Measures taken to deal with new diseases were intensified about the same time as the World Health Organization (WHO) classified COVID-19 as a pandemic. Some of these measures were repressive, meaning there was a forceful intervention by the state in peoples’ life in order to push back the pandemic, such as travel restrictions and banning of visits to nursing homes and gatherings of more than 50 people. People aged 70 or more were recommended to stay isolated. The upper secondary schools were closed. Other responses were based on confidence in citizens’ self-governing behavior, a kind of self-induced intervention to address the pandemic based on trust. However, one of the most noticeable components in the Swedish strategy to handle the pandemic was keeping preschools and comprehensive schools open based on self-governing behavior. There were also supporting measures directed to the schools. All three kinds of measures—repressive, self-governing, and supporting—can be seen as components in the making of an assemblage triggered by the COVID-19 pandemic (Table 1).

This section deals with how strategic responses to the pandemic came to embrace schools, as these were represented in daily newspapers and teachers’ trade union journals and homepages.^6^ When the question whether to close schools was raised, the most salient argument was that daily care and a safe environment at preschools and schools were prerequisites for parents’ participation in essential functions in society. For instance, it was estimated that of all children in preschool and comprehensive schools, around 10% had parents working in the health care sector.^7^ Another argument was that daily routines to go to school and receive care and daily meals protected many children from insufficient conditions at home.

The schools were expected to follow the directives of the Public Health Agency, which demanded handwashing with soap and water, added posters on walls with instructions on how to handwash, and ensured that there is access to disinfectants when water for handwashing was unavailable. In all activities, distances between teachers and pupils had to be maintained in classrooms, dining halls, and other spaces; gatherings should be avoided, and recreation and teaching activities should take place outside, if possible. Finally, handles, surfaces, screens of laptops, and iPads should be cleaned at least once a day.^8^

The local authorities (municipalities and organizers of independent schools) mandated schools to make their emergency plans adaptable in order to keep these flexible when important societal functions did not work. For instance, the following aspects were covered:

- how to organize the teaching if many teachers were absent during a longer period
- how the staff could get to school if public transportation is stopped
- how to organize the supply of food and sanitary items^9^

Schools should also be prepared to close rapidly if many teachers were absent or if it was necessary to avoid the further spread of the virus in the district.

The School Law (SFS 2020:148) (see Table 1) was changed to make it possible for the government and the local authorities to close schools. However, it was considered a last resort. Some schools closed for shorter periods when many teachers were absent and when continued teaching was impossible.^10^ Many followed the advice to stay at home when feeling ill, and, particularly during the first month of the pandemic, the number of absent students and teachers was high. Teachers’ trade unions expressed concerns that teachers were exposed to contagion at work, but according to statistics presented by the National Health Agency, there was no evidence that teachers were more exposed than other groups were.^11^

After Easter holidays in the middle of April, when many children still did not attend school, the Minister of Education emphasized that there were no medical reasons to keep healthy children at home.^12^ The media reported that school absence was around 25–30% among teachers and students. In the beginning of May, the level of absent pupils was twice as usual, around 15%^13^. As the number of absent students increased, the National Agency for Education emphasized that school attendance was mandatory in accordance with the School Law, and the possibility of fining those parents who, for no other reasons than worries, kept their children from school was discussed.^14^ Schools made efforts to convince parents who still kept their children at home, and school health services were engaged at the end of the semester to talk with these parents.^15^

Trade unions argued that many schools had a shortage of teachers and large classes, and they were worried about further downsizing in many municipalities. Teaching online was a new experience and a challenge for many upper secondary teachers. In preschools and schools, teachers had to replace absent colleagues and give non-present pupils support online. An inquiry made by one teachers’ trade union reveals that 20% of the teachers said that they had to provide absent pupils with assignments and teaching online in addition to their usual tasks.^16^

However, the question on whether schools should be open or not remained at the agenda. At the end of April, a revision of school legislation introduced distance teaching in compulsory school, aiming to temporarily implement distance teaching in order to cater to the needs of teachers and pupils who had to stay at home in line with the recommendations of the National Health Agency. It was introduced as enhanced flexibility by the Minister of Education but was criticized by teachers’ trade unions, which feared that it would justify local authorities offering online teaching to an extent that would widely exceed the goals in the government’s decision.^17^

In the measures taken to maintain presence at schools, intricate collections of interactions become visible. At different levels, the Ministry of Education, the National Agency for Education, local authorities and schools, school teachers, and school health services were intervening with the available tools in order to keep students in schools.

## Responses from the National Agency for Education

Regarding the pandemic, the National Agency for Education acted through information, guidelines, and recommendations to schools and local authorities. Health information on how to behave well, built on guidelines from the Public Health Agency, was published on its webpage.

During spring, the local authorities were given the right by the government to make exceptions from the regulations concerning the design, scope, and location of education (see Table 1). The National Agency for Education provided support,^18^ including a short checklist in case of a school closure, directed to the local authorities and with reference to the Civil Contingencies Agency. The topics concerned communication plans and maintaining important societal activities in case of a school closure. Following the Swedish constitution (see Table 1), the local authorities have the responsibility of providing schools with support. They were considered parts of the assemblage as co-agents in the crisis based on their unique areas of responsibilities. All this could be interpreted as signifying a self-governing attitude based on mutual trust.

At the end of April, the government specifically addressed the National Agency for Education, to form a commission to support and assist schools in their efforts to stay open during the pandemic (see Table 1) (Ministry of Education 2020). The commission follows the line of command between the government and public agencies. Actions taken as a response to the new commission should be based on the expertise of the authorities. This indicates a further connection with the assemblage regulating the pandemic and including an internalization of other agencies’ *grammar *or elements, particularly the scientific basis of the Public Health Agency.

The constitution of Sweden stipulates that government decisions are collective cabinet decisions, resulting in directives that semi-autonomous administrative agencies have a duty to obey. This role is underlined by the freedom of agencies to interpret and apply laws in individual cases (SFS 1974:152; Sveriges Riksdag 2016). That is, the parts of the state are not bound by any hierarchical position vis-á-vis one another and somehow enjoy the freedom to act. The school not only has a knowledge assignment but also a childcare assignment. The National Agency for Education handled the risk by referring to compulsory school’s child care assignment, transferring the risk to school organizers, municipalities, and private individuals, which, in practice, meant the teachers.

In the next section, we broaden the picture and include other agents to make visible the flow of events and actions involved in the making of an assemblage.

## The welfare state constitution and addressing a pandemic

The Public Health Agency was positioned as the center for Swedish monitoring of the pandemic, and it continuously provided societal actors and the general public with relevant information. If the Public Health Agency was the knowledge organization based on scientific expertise, then the National Agency of Education was the supporting agency, translating or implementing directives given to them. The evolving pandemic in Sweden soon appeared in mass-media as numbers and curves, derived from the Public Health Agency. First the numbers in intensive care rapidly increased in March and early April and then decrease to very few cases at the end of July 2020.^21^ This decrease was often related to improved behavior in accordance with the pedagogical program presented below.

The Public Health Agency was not unprepared when the pandemic broke out; manuals for pandemic control had been in place earlier (See e.g. National Board of Health and Welfare (2005)), in the aftermaths of earlier global pandemics and had been discussed and revised within networks of local, regional, national, and global actors. Regions and municipalities had developed their own plans in connection with national initiatives.

A pedagogical program for the education of the population started early. It was prepared long before the current pandemic broke out; the architects were the Public Health Agency in close collaboration with the WHO and other national and international actors in the field of contagious diseases. The pedagogical program was repeated during daily press briefings and intensively communicated in newspapers, on TV, official homepages, and almost everywhere in society. The educational content was directed toward individual behavior. People were instructed how to protect themselves and others from being infected. Everyone should behave well or take a personal responsibility to follow the recommendations. The main content of the pedagogical program was as follows:

- Stay at home if you are feeling unwell.
- Keep your distance from other people (1.5–2 meters).
- Wash your hands frequently with soap and water.
- Avoid social events.

This program was a kind of self-governing measure that covered all citizens, including teachers, students, and their parents. As mentioned, it was present in the everyday life of schools. Information from all public agencies involved should be unified, following the strategy of the pandemic plan.

In the middle of March, it was still not evident whether comprehensive schools should remain open. After a few days of preparation and just a few days after the WHO had declared COVID-19 as a pandemic, the School Law^22^ was changed (see Table 1) to make it possible to close a school. Such a decision might very well have been made during the following weeks, at least if we look at it from an outsider perspective. Lockdowns and school closures as signs of repressive responses were reported all over Europe.

Could the decisions made in the middle of March and that made it possible to close comprehensive schools be considered a kind of panic measure in the management of a sudden and unexpected societal crisis? Most likely not. In their most recent manual for pandemic control, published in December 2019, the Public Health Agency (2019) mentions the possibility of closing schools as a non-pharmaceutical measure that also follows WHO manuals for pandemic control.^23^

In March, all the necessary formal preparations for the closure of comprehensive schools were taken in Sweden. Following the Swedish welfare state, the new legislation transferred responsibilities to those with comprehensive tasks for the schools—the municipalities and independent schools. Therefore, they also became agents and constituents of an assemblage in their response to the pandemic and had to act accordingly. A school could be closed by local educational authorities but only under the following conditions:

- If many staff are absent because of the pandemic (i.e., a disease listed in the Communicable Diseases Act (Government Bill 2019/20, p. 144)), and, therefore, it is not possible to continue teaching
- When preventing the spread of disease listed in the Communicable Diseases Act, in consultation with the County Medical Officer of Communicable Diseases
- If the school is located in an area that the Public Health Agency has decided should be locked down according to the Communicable Diseases Act
- Because of a recommendation from the Public Health Agency that concerns the school sector

We will emphasize here the interwinding between schools (absent staff), local authorities (responsible agents), legislation (Communicable Diseases Act and the School Law), regional medical officers (experts), and the Public Health Agency (experts), which also illustrates how the Swedish constitution works and, taken together, becomes a forceful regulator of an assemblage for managing a pandemic.

## The Swedish welfare state constitution

Based on Esping-Andersen’s three types of welfare state capitalism, Sweden is described as a social-democratic welfare regime, meaning it has relatively high taxation and distribution of welfare resources of high standards, irrespective of income (Esping-Andersen 1990). Local self-governance, including economic responsibilities for the provision of welfare services, is based on political and administrative decentralization at the regional and local levels. This kind of reasoning also covers schools. An understanding of the flow of events and how parts become interwoven in the making of an assemblage must therefore embrace the Swedish welfare state constitution as a foundation. A welfare state constitution refers here to how the state is governed, including political decision making, legislation, and public administration, which are based on fundamental rules, principles, and procedures.

What might stand out in relation to many other countries and that most likely has had a strong impact on how the COVID-19 pandemic has been managed in Sweden in relation to schools is the long tradition of public agencies that are organizationally separated from the ministries. In a so-called letter of regulation, the government decides each year about the budget and how the agency in question should work. Public agencies can be rather large compared with the ministries (Lægreid 2017). However, the government’s office does not lack political capacity, and politicians can always make amendments to the letter of regulation. The task of public agencies is to promote the politics of the government, but they are expected to act independently.

Today, public trust in public agencies is much higher than trust in politicians (Lindblad et al. 2018). This trust was established through a series of political reforms in the 19th century that came to address massive corruption in the political administration of Sweden. Educational reforms and well-educated civil servants played a vital role in indirectly defeating corruption (Rothstein 2011).

Consequently, following Swedish administrative traditions, the COVID-19 crisis is not only managed by the government but also by autonomous regions, the local authorities, and public agencies. In addition to this, management by results has mainly had a focus on processes and procedures centrally, regionally, and locally but not on coordinating activities, placing teachers as the frontline agents between the state and citizens (Goodson and Lindblad 2011; Houtsonen and Wärvik 2009). Taken together, Swedish administrative traditions give the impression of a rather complex net of decision making based on independent agents of different kinds. Public agencies work out recommendations on how laws and regulations should be applied based on their areas of expertise. These recommendations are guidelines on how to act. Even if a recommendation from a public agency is not legally binding, following it is necessary, as such recommendations are founded on laws, ordinances, and regulations.

## Concluding discussion

“We have managed to keep the schools open,” said Dr. Tegnell, Sweden’s state epidemiologist. Why was this the response to the COVID-19 pandemic in Sweden? What were the implications of this response so far?

First, what stood out as significant in a no-vaccine COVID-19 assemblage was the general principle of education of the people into a self-governing attitude. Decreasing numbers of persons infected or hospitalized were presented as due to appropriate and solidary behavior by the people, whereas the lack of social distancing was presented as dangerous and to be avoided. To our understanding this was the basic idea of education of the population in competent behavior in order to manage the pandemic.

Second, we identified a large collection of interacting agents in the making of strategic responses that include not only institutions but also laws and ordinances, regulations and recommendations, pandemic manuals and emergency plans, curves, statistics, newspapers, and media channels. All these were brought together by the purpose of handling the implications that the crisis brought upon education. This collection is vital to capture in order to understand the working of the Swedish case.

Third, the school system was part of the general principle responding from its position as a non-pharmaceutical intervention on the pandemic; its responses were rooted in the actions of a large range of agents. From the outside, it could look like a unified and well-working machinery:

- A national pedagogical program regarding hygiene and distancing in times of a pandemic was developed and implemented in schools and in society as a whole.
- The pandemic materialized as curves that was going up and down to show compliance or non-compliance with the pedagogical program.
- Children and teachers learned how a pandemic functioned and could behave well in times of a pandemic.
- Schools could stay open, and children could go to school.

It all looked very simple and easy. The foundation was solid, and the pandemic triggered changes in the Communicable Diseases Act and the School Law. Public agencies worked together and coordinated their efforts, with the Public Health Agency being the touchstone as a knowledge organization and the national Agency for Education as a supporting organization. It was about generative and interactive strategic responses in a complex web of agents associated with one another when controlling the pandemic *and* triggered by the pandemic. In short, an education assemblage was created as a stabilizing order.

Fourth, at the same time this was not a machine. The pandemic created disorder in society and in education in several ways. The manifold of interacting agents out of their normality turned visible in that everything was not working smoothly, for instance, between national and local authorities and schools. Weaknesses also become visible in the responses of teachers as frontline agents. Following the Swedish welfare state administration and ideas of management by results introduced in the 1990s, a main responsibility was given to these frontline agents. It was the schools and ultimately the teachers—situated between the state and citizens—who had the responsibilities for handling the pandemic. They were expected to solve the problems in the daily work of schools. Students could, for instance, refuse to comply and stay at home even if their school was open and they were healthy. Persons with even very mild symptoms should stay at home, meaning that schools, students, and teachers had to reorganize their daily work to keep activities going on. This was an agency based on trust, but it could pose risks for everyone involved. A call for central governance was potentially present in the making of responses based on the key question of whether the curves were going up or down. Even so, the responsibility was pushed back, with the pedagogical program, recommendations, local emergency plans, and the curves as mediating agents, following a logic of the Swedish welfare state constitution. Added to this is the observation that scientific expertise and its uncertainties were important ingredients in public discourses.

Although our inquiries are carried out long before the pandemic was over, we would like to state a few notions related to knowledge on education and society. One, considering studies on education and social reproduction, we presented an assemblage of different agents, actions, and interactions that sometimes challenge predominant patterns of power and sometimes stabilizes these patterns (such as Bourdieu and Passeron 1990 and Altvater and Huisken 1971). This we consider a way to further knowledge on education in societal contexts. Two, we noted that education turned to be vital in an overarching strategy to deal with the pandemic in Sweden in terms of trust in people and their governmentality (Foucault and Ewald 2003). These conclusions are based on the extraordinary event that we are studying, but we must note that historically, schooling is often developed during extraordinary events, such as saving a population in misery (Hunter 1994), and in contested responses to societal changes (Durkheim 2013, on the evolution of education in France, and Katz 1992, on progressive reforms in Chicago).

To end, we found in our case ambitions to produce alternative education approaches such as distance education and teaching via internet. We strongly recommend that these alternatives are analyzed as broad educational measures—not only in terms of efficiency, but also how they function as instruments for educating culturally competent persons in our time!

## Footnotes

1 https://www.youtube.com/watch?v=xh9wso6bEAc.

2 The coronavirus disease 2019, or COVID-19, emerged in 2020 and turned into a pandemic that resulted in a global crisis demanding responses from countries all over the world.

3 Culturally competent persons who are able and willing to follow recommendations in terms of social distancing and personal hygiene, in other words, a product of an education subsystem (Luhmann and Schorr 2000).

4 Hunter’s (1994) historical analysis has a Foucauldian twist that is of interest here.

5 We are referring here to the distinction between the front stage and the back stage in presenting oneself according to Goffman (1956).

6 References to news reporting are retrieved from the database Mediearkivet. The search items corona* AND school*, and covid* AND school* were used for four daily newspapers: Dagens Nyheter, Svenska Dagbladet, Göteborgs-Posten, Sydsvenska Dagbladet, TT Nyhetsbyrån and the two tabloids Aftonbladet and Expressen. Also used as sources were the teacher trade union journals Skolvärlden and Lärarnas tidning, as well as their home pages.

7 Aftonbladet 17 March (2020); Svenska Dagbladet 21 March (2020).

8 https://www.folkhalsomyndigheten.se/smittskydd-beredskap/utbrott/aktuella-utbrott/covid-19/verksamheter/information-till-skola-och-forskola-om-den-nya-sjukdomen-covid-19/.

9 https://mitti.se/lansnyheter/stockholms-skolor-pandemiplan/.

10 https://skolvarlden.se/artiklar/flera-grundskolor-stanger-helt-eller-delvis-efter-pask.

11 https://skolvarlden.se/artiklar/lagre-risk-att-larare-smittas-andra-yrkesgrupper.

12 Svenska Dagbladet 2 April (2020).

13 https://www.svt.se/nyheter/inrikes/skola-3.

14 TT Nyhetsbyrån 8 May (2020).

15 Sydsvenskan 17 June (2020).

16 https://skolvarlden.se/artiklar/corona-slaggan-sa-drabbas-skolan-ekonomiskt-host.

17 Skolvärlden 24 April 2020.

18 https://www.skolverket.se/regler-och-ansvar/coronaviruset-och-covid-19---regler-for-skolor-och-forskolor/regler-for-forskolor-och-skolor-som-haller-oppet-eller-behover-stanga-pa-grund-av-coronapandemin.

21 https://www.folkhalsomyndigheten.se/smittskydd-beredskap/utbrott/aktuella-utbrott/covid-19/statistik-och-analyser/bekraftade-fall-i-sverige/.

22 SFS (2010:800).

23 https://apps.who.int/iris/handle/10665/259893.
